# Some Experiences of a Bird Collector

**Published:** 1911-05-27

**Authors:** 


### The Practitioner's Relaxations.

### SOME EXPERIENCES OF A BIRD COLLECTOR.

The hobby of collecting and setting up stuffed birds has given place to the far more humane and doubtless more fascinating pursuit of obtaining photographs of the living birds. Yet the older hobby afforded opportunities for the display of patience, physical endurance, occasionally, perhaps, adventure, by no means infrequently pleasurable excitement, though at other times doubtless disappointment. The hobby did not, however, consist merely of the pursuit of desirable specimens. The bird-collector was first a bird-lover and more time was spent in observation than in pursuit, and not only did every variety of song, of call, or of alarm-note become familiar, but every bird became known by movement on the wing, amongst the branches, or on the ground. The peculiar little ways of every individual bird lent both an artistic and scientific interest to the setting up of any specimen, and the amateur taxidermist did his best to impart something of the air of life which he had learned to recognise and to admire in the field, the wood, or on the sea-shore.

### Bird Collecting.

Small birds I used to shoot with a walking-stick gun, and I have been guilty of obtaining specimens with this instrument in situations, it is to be feared, which placed me within the risk of suffering at the hands of the law. With this small weapon I once secured a dunlin, a bird which those to whom the bird is familiar would consider to be difficult to approach sufficiently near to shoot without a gun possessing a moderate range. The circumstances, however, were somewhat peculiar and resulted in a surprise and, it may be added, a temporary disappointment. On a beautiful summer evening in July, a few days before the date upon which the law allows the shooting of wildfowl to commence, I was sitting near the edge of a grassy mere listening to the cries of various species of waders and now and then getting sight of some in their whirling flight. My inseparable companion, my walking-stick gun, was with me. Quietly and unexpectedly a male dunlin still possessing the black breast of its summer plumage walked, only about twenty yards away, from behind some tufts of grass. I wished for a specimen in this plumage and shot it. The sound of my shot alarmed three or four other dunlins which had been hidden from view behind the same tufts of grass. It was not, however, these which attracted my attention. Rising with them were two curlew sandpipers, my first and only meeting with these elegant reproductions of the curlew in miniature, both of them sufficiently close to enable me to recognise nearly every feature and to see that they possessed the greater part of their reddish chestnut summer plumage. Here pleasure was mixed with disappointment. Had I not shot the dunlin—at least, so I surmised—a curlew sandpiper might have walked into view and within range of my walking-stick gun. The next day these wanderers might be many miles away, and I never expected to see them again; and I did not see them alive, but on the second day of the shooting season on my return home to my lodgings I found those two birds left there by a gunner whose punt I had hired. Although, naturally, the birds shot by others have not the interest of those recognised, pursued, and secured by oneself, an uncommon bird is always welcome from almost any source. A few days later another gunner brought me a bird which surpasses in neatness, elegance, and beauty of colouring the curlew sandpiper. This bird was the red-necked phalarope, a bird now known to nest in the northern part of these islands and of which Mr. Kearton gives a charming little photograph in one of his books, swimming near its nesting haunt.

Yet this particular holiday did not fail to provide me with something of the reward which is hoped for as the result of individual effort. Towards the end of August, when out punting, two small wading-birds rose some distance off uttering a note unknown to me. This was in the morning. All the rest of that morning and all the following afternoon I punted and walked in the hope of meeting with the strangers, without success. Up before daybreak the next morning, I searched mere and marsh again, but still without success. Towards the end of the day, however, the two birds sought for rose at no great distance from me uttering their peculiar note. I marked the spot at which they settled, followed, and in a few minutes had procured both. They proved to be Temminck's stints.

The Blue-Throated Warbler.

Short accounts of the interest and pleasure of meeting with birds for the first time either in various parts of this country or abroad could be mentioned. The bullying tactics of the Richardson's skua, the difficulty of seeing the brilliant golden oriole, or the peculiar evolutions of the still more brilliant bee-eater all have some charm to the memory. But more in keeping with the short accounts given above is my experience of meeting with the blue-throated warbler. It is scarcely necessary to mention that numerous small birds other than waders pass down our east coast during the autumn migration. Even the tiny gold-crest crosses the North Sea in thousands, a bird so delicate that the son of the man whose punt I had hired (and obtained me the curlew sandpipers) told me that the shock of a stone thrown into bushes where they were resting was sufficient to kill them. Apparently, for some reason, a lady wished for a few of these birds, and the boy told me that he had obtained several in the way above mentioned as they sat in their hundreds in the bushes after arrival. He considered that they were killed merely by being hit by the twigs. To speak, however of the blue-throated warbler. When crossing over a wide estuary in a boat during a thick fog with two of my brothers, a bird, evidently tired by a long flight, settled on the gunwale of our boat. It was a bird with no particularly beautiful colouring upon it, but it was a stranger. Although it had settled, the close proximity of man was soon recognised and it took to flight again. As the bird slowly flew towards the shore it displayed a reddish band on the upper half of the tail and I knew that it was a blue-throated warbler. The beautiful plumage of the adult bird was not yet present. In spite of the fact that my desire for acquisition was aroused, and I spent the following day searching the neighbourhood of the shore for miles, but without result. Two days later I again sailed to the opposite shore and there was rewarded. I obtained three specimens; one like the bird which had settled upon the boat, a bird of the year, but the other two were birds of the second year, birds in which the beautiful blue breast was partially but, unfortunately, not fully developed.

There is possibly always some degree of pleasure in the obtaining of any object upon which the mind has been set. In the collecting of birds there may not be only the pleasure of acquiring something wished for, but there may be some satisfaction to the sporting instinct. It is undoubtedly a pleasure to bring down a fast-flying bird almost before there has been time to realise that it has made its appearance. But to the bird-lover, as one sees it lying dead before a feather has been displaced by the touch of a human hand, the pleasure may be counterbalanced by an equal degree of pain. Unfortunately, to photograph all species of birds is virtually impossible. Yet the aim, although confronted with difficulties, is free from the risk of wounding sentiment born of admiration for the beautiful.

